# Risk assessment and triage strategy of cervical cancer primary screening on HPV integration status: 5-year follow-up of a prospective cohort study

**DOI:** 10.1016/j.jncc.2024.08.001

**Published:** 2024-10-16

**Authors:** Xun Tian, Danhui Weng, Ye Chen, Yi Wang, Xiao Li, Xin Wang, Chen Cao, Danni Gong, Zhen Zeng, Qiongyan Wu, Xueqian Wang, Peng Wu, Lu Fan, Qinghua Zhang, Hui Wang, Zheng Hu, Xiaodong Cheng, Ding Ma

**Affiliations:** 1 Department of Gynecological Oncology, Tongji Hospital, Tongji Medical College, Huazhong University of Science and Technology, Wuhan, China; 2 National Clinical Research Center for Obstetrics and Gynecology, Cancer Biology Research Center (Key Laboratory of the Ministry of Education), Tongji Hospital, Tongji Medical College, Huazhong University of Science and Technology, Wuhan, China; 3 Department of Obstetrics and Gynecology, Academician expert workstation, The Central Hospital of Wuhan, Tongji Medical College, Huazhong University of Science and Technology, Wuhan, China; 4 Department of Gynecology, The Sixth Affiliated Hospital, Sun Yat-sen University, Guangzhou, China; 5 Biomedical Innovation Center, The Sixth Affiliated Hospital, Sun Yat-sen University, Guangzhou, China; 6 Department of Obstetrics and Gynecology, The Affiliated Panyu Central Hospital of Guangzhou Medical University, Guangzhou, China; 7 Department of Epidemiology and Biostatistics, School of Public Health, Tongji Medical College, Huazhong University of Science and Technology, Wuhan, China; 8 Department of Gynecologic Oncology, Women's Hospital, School of Medicine, Zhejiang University, Hangzhou, China; 9 Department of Gynecology and Obstetrics, Union Hospital, Tongji Medical College, Huazhong University of Science and Technology, Wuhan, China; 10 Maternal and Child Health and Family Planning Service Center of Longyou County, Quzhou, China; 11 Department of Gynecologic Oncology, Zhongnan Hospital of Wuhan University, Wuhan, China

**Keywords:** Human papillomavirus, Cervical cancer screening, HPV integration, Colposcopy, Cervical intraepithelial neoplasia

## Abstract

**Objective:**

We investigated the relation between man papillomavirus (HPV) integration status and the immediate risk of cervical intraepithelial neoplasia (CIN), as well as the triage strategy based on HPV integration test.

**Methods:**

4086 women aged 20 to 65 years in China were enrolled in 2015 for a prospective, population-based, clinical observational study to evaluate the triage performance of HPV integration. Cervical exfoliated cells were collected for HPV testing and cytologic test. If high-risk HPV was positive, HPV integration test was performed at baseline, 2-year and 5-year follow-up.

**Results:**

At baseline, HPV integration was positively correlated with the severity of cervical pathology, ranging from 5.0% (15/301) in normal diagnosis, 6.9% (4/58) in CIN1, 31.0% (9/29) in CIN2, 70% (14/20) in CIN3, and 100% (2/2) in cervical cancer (*P* < 0.001). Compared with cytology, HPV integration exhibits comparable sensitivity and negative predictive value for the diagnosis of CIN3+, higher specificity (92.8% [90.2%–95.4%] vs. 75.5% [71.2%–79.8%], *P* < 0.001) and higher positive predictive value (36.4% [22.1%–50.6%] vs. 15.2% [8.5%–21.8%], *P* < 0.001). HPV integration testing strategy yielded a significantly lower colposcopy referral rate than cytology strategy (10.7% [44/410] vs. 27.3% [112/410], *P* < 0.001). The HPV integration-negative group exhibited the lowest immediate risk for CIN3+ (1.6%) and accounted for the largest proportion of the total population (89.3%), when compared with the normal cytology group (risk, 1.7%; proportion, 72.7%).

**Conclusion:**

As a key molecular basis for the development of cervical cancer, HPV integration might be a promising triage strategy for HPV-positive patients.

## 1 Introduction

Human papillomavirus (HPV) infection is known as the major cause of cervical cancer, leading to nearly 600,000 new cases and 340,000 deaths per year.^1^ The World Health Organization guideline recommends using HPV DNA detection as the primary screening for the general population.^2^^,^^3^ Although HPV infection is prevalent,^4^ most cases are transient due to rapid immune clearance.^5^, ^6^, ^7^ Only a small proportion persists and progresses to cervical cancer.^8^^,^^9^ Thus, triage biomarkers for HPV-positive women are necessary to distinguish those at increased risk of cervical cancer from those who will spontaneously clear the infection, thus avoiding unnecessary colposcopy referrals and overtreatment.^10^

Cervical cytology using Thinprep cytologic test (TCT) was initially recommended as a triage method for HPV-positive women.^11^^,^^12^ However, this approach requires systematic professional training, which is often lacking in developing countries and other resource-limited settings. Furthermore, the absence of uniform standards for cytological interpretation hampers the comparability and reproducibility of results. To overcome these limitations, several alternative triage biomarkers have been proposed, including p16/Ki-67 staining,^13^ methylation status,^14^ and the OncoE6 test.^15^ Despite their promise, these biomarkers have not yet been widely adopted due to the lack of standardized protocols, inter-laboratory variability, and cost considerations. Therefore, the development of an objective triage strategy will be advantageous in the future.

HPV integration is a pivotal event in which HPV E6/E7 oncogenes are inserted into the host genome, thereby promoting persistent HPV infection and facilitating cervical carcinogenesis and progression.^16^, ^17^, ^18^ With the advancement of sequencing technology, next-generation sequencing (NGS) has become a reliable method for assessing HPV integration status including HPV status and integration signatures.^19^ Several reports have demonstrated that the frequency of HPV integration events is significantly higher in precancerous lesions and cancers compared to normal cervix, highlighting the potential of HPV integration as a risk stratification biomarker for cervical cancer.^20^^,^^21^ We conducted a prospective, longitudinal study to assess the clinical effectiveness of HPV integration status as a risk stratification biomarker for cervical cancer screening.

## 2 Patients and methods

### 2.1 Study design and patients

**Fig. 1:**
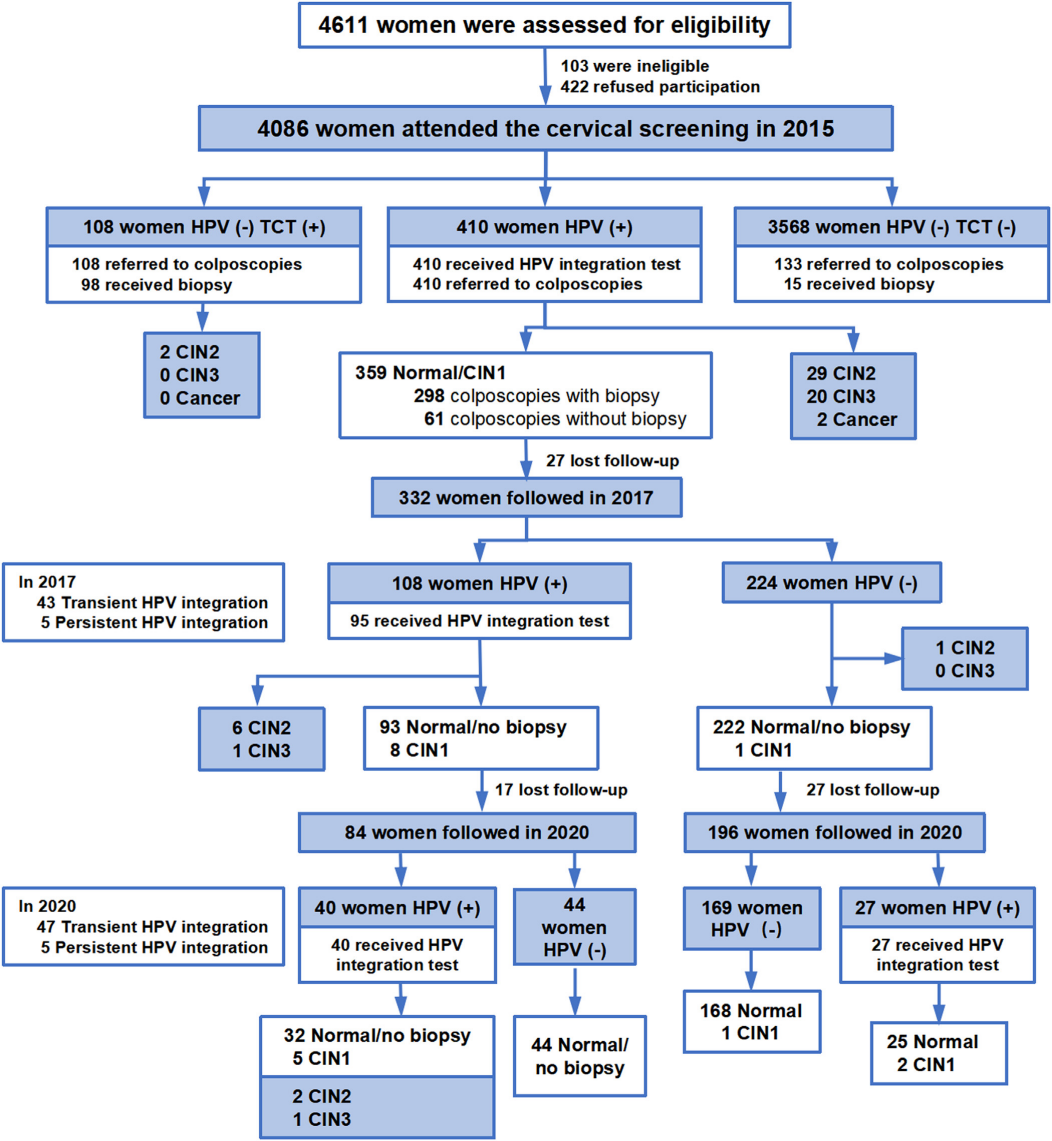
Study population enrollment flow diagram. Abbreviations: HPV, human papillomavirus; TCT, Thinprep cytologic test; CIN, cervical intraepithelial neoplasia.

The study was launched in April 2015, in which 4611 eligible women between the ages of 20 to 65 years, residing in Longyou County in Quzhou City, Zhejiang Province, China, were invited to attend the cervical cancer routine screening. The detailed inclusion and exclusion criteria for participants were described in a previous study.^22^ Finally, 4086 women were enrolled in the study. The recruitment is depicted in Fig. 1. In HPV-positive patients, 6.6% (27/410) of individuals were lost to follow-up.

Women who were tested with abnormal screening results (HPV-positive or atypical squamous cells of undetermined significance [ASC-US+]) received colposcopy, with a directed cervical biopsy performed if necessary. Patients with abnormal screening results were recalled for screening in the next round. Besides, all of the participants with positive HPV test results at baseline were recalled for HPV integration tests in 2017 and 2020, respectively, and the entire screening procedures remained consistent with the 2015 baseline screening tests. To facilitate the observation of dynamic changes in HPV infection and integration status, we repeated the HPV integration test at 2-year follow-up and 5-year follow-up, given that HPV infection frequently clears up spontaneously within 1-2 years.^5^^,^^6^ Patients diagnosed with CIN2 or worse (CIN2+) lesions were referred for corresponding treatment by the American Society for Colposcopy and Cervical Pathology (ASCCP) consensus guidelines.^23^

Before enrollment in the study, all participants provided written informed consent. The research protocol was reviewed and approved by the ethics committee at Tongji Hospital, Huazhong University of Science and Technology with registration number TJ-C20150802. The study protocol was registered under ClinicalTrials.gov (number NCT02576158).

### 2.2 Screening methods

Cervical exfoliated cells were stored in a medium for both high-risk HPV types test (Cobas, Roche Diagnostics, Indianapolis, IN, USA) and liquid-based thin-layer cytological assay (SurePath, Becton Dickinson, Franklin Lakes, NJ, USA). Fourteen high-risk HPV types, including 16, 18, 31, 33, 35, 39, 45, 51, 52, 56, 58, 59, 66, and 68, were detected according to the manufacturer's instructions. The liquid-based thin-layer slide was prepared for the Thinprep cytologic test. Cytological results were reported according to the Bethesda system. The cytologic results were mainly divided into the following categories: intraepithelial lesion/malignant lesion cell (NILM), atypical squamous cells of undetermined significance (ASCUS), low-grade squamous intraepithelial lesion (LSIL), atypical squamous cells-cannot rule out high-grade squamous intraepithelial lesion (ASC-H), atypical glandular cells (AGC), high-grade squamous intraepithelial lesion (HSIL), and cervical cancer cells. Histological diagnoses are determined based on the CIN classification.^24^^,^^25^

### 2.3 HPV DNA integration testing by HPV capture sequencing technology

Specimens that confirmed the presence of high-risk HPV were subjected to an HPV integration test. Detection of HPV integration was performed using HPV Capture Hybridization and the next-generation sequencing (NGS) analysis.^26^ Human reference genome and the whole genomic sequence of 14 types of high-risk HPV (hrHPV) were obtained from NCBI (https://www.ncbi.nlm.nih.gov/). hrHPV accession numbers were as follows: NC_001526.4(HPV16), AY262282.1(HPV18), HQ537687.1(HPV31), HQ537688.1(HPV33), HQ537730.1(HPV35), LR862071.1(HPV39), EF202167.1(HPV45), LR862072.1(HPV51), HQ537751.1(HPV52), EF177181.1(HPV56), HQ537777.1(HPV58), EU918767.1(HPV59), EF177191.1(HPV66), and FR751039.1(HPV68).

NGS sequencing was performed using Illumina NovaSeq PE150. After removing low-quality and duplicate reads, clean reads were obtained for subsequent analysis. Clean reads were mapped to human (NCBI build 37, hg19) and HPV genomes. Chimeric paired-end reads (partial alignment with human genome, partial alignment with HPV genome) were reserved for further assembly. All the data were processed and analyzed by Survirus pipeline and the breakpoints with support reads ≥10 were retained.^27^^,^^28^

### 2.4 Statistical analysis

The primary endpoint was the incidence of histologic CIN3+. The second endpoint was the incidence of histologic CIN2+. Women with HPV-negative and normal cytology results were considered free of disease in the present study. Women with only once detected for HPV integration were defined as the transient integration group. Women with ≥ twice detected for HPV integration were defined as the persistent integration group. Stata/SE 15.0 (StataCorp) was performed for statistical analysis. Continuous variables with normal distribution were presented as mean ± standard deviation (SD). Incidence of HPV infection, abnormal cytology, and HPV integration were calculated as proportions. Sensitivity, specificity, accuracy, positive predictive value (PPV), and negative predictive value (NPV) were presented in the percentage form. Cochran–Armitage test was used to assess linear trends when exploring the relationship among HPV integration, cytology, and pathology. Differences in positivity, sensitivity, and specificity were evaluated using an exact McNemar χ^2^ and differences in PPV and NPV were assessed using the method developed by Leisenring et al.^29^ using the R package, DTComPair (R Foundation for Statistical Computing). Chi-square or Fisher's exact test was performed for group comparisons of other categorical data. *P* value <0.05 was considered statistically significant. All tests were performed as two-sided.

## 3 Results

### 3.1 Baseline characteristics of study population

Fig. 1 illustrates the enrollment process and schedule for the baseline and follow-up. A total of 4086 women were deemed eligible and enrolled in the study at baseline. The mean (±SD) age of the participants was 46.2 ± 8.6 years.

The characteristics of the participants at baseline are presented in Supplementary Table 1. In the baseline screening round, there were 410/4086 (10.0%) women tested positive for hrHPV, and 44 women were confirmed to be HPV integration positive (44/410, 10.7%). Among the 108 (26.3%) women who were tested positive for HPV16/18, 27 (25%) women were confirmed to be HPV integration-positive. This frequency was significantly higher compared with non-HPV16/18 women, among whom only 17 out of 302 (5.6%) were HPV integration-positive (*P* < 0.001).

Among 410 HPV-positive women, a biopsy showed that 108 women had abnormal pathological diagnoses, including 58 cases with CIN1, 29 cases with CIN2, 20 cases with CIN3, and 2 cases of cervical cancer. As illustrated in Supplementary Fig. 1 and Supplementary Table 2, the proportion of HPV integration-positive cases gradually increased from patients with normal diagnosis (5.0%, 15/301) to CIN1 (6.9%, 4/58), CIN2 (31.0%, 9/29), CIN3 (70%, 14/20), and cancer (100%, 2/2), demonstrating a statistically significant trend (*P*<0.001). The proportion of TCT abnormalities increased from patients with normal diagnosis (18.9%, 57/301) to CIN1 (39.7%, 23/58), CIN2 (51.7%, 15/29), CIN3 (80%, 16/20), and cancer (50%, 1/2), showing an upward trend (*P* < 0.001).

### 3.2 Triage performance of HPV integration for detection of CIN2+/CIN3+ in HPV-positive patients

In the context of 410 hrHPV-positive patients, the baseline cross-sectional analysis illustrates that HPV integration exhibited comparable sensitivity and NPV to TCT for diagnosis of CIN3+, higher specificity (92.8% [90.2%–95.4%] vs. 75.5% [71.2%–79.8%], *P* < 0.001**)** and higher PPV (36.4% [22.1%–50.6%] vs. 15.2% [8.5%–21.8%], *P* < 0.001) (Table 1). HPV integration testing strategy yielded lower colposcopy referral rate than TCT testing strategy (10.7% [44/410] vs. 27.3% [112/410], *P* < 0.001).

**Table 1:** Triage performance for detection of CIN2+/CIN3+ in 410 HPV-positive women and 108 HPV16/18-positive women.

	HPV-positive women (n = 410)			HPV16/18-positive women (n = 108)		
	HPV Integration	TCT	*P* value	HPV Integration	TCT	*P* value
Positivity, % (No./total patients)	10.7 (44/410)	27.3 (112/410)	<0.001^a^	25 (27/108)	32.4 (35/108)	0.186^a^
Colposcopy referral rate, % (No./total patients)	10.7 (44/410)	27.3 (112/410)		25 (27/108)	32.4 (35/108)	
Detection of CIN3+						
Sensitivity, % (95% CI)	72.7 (54.1–91.3)	77.3 (59.8–94.8)	0.739	77.8 (58.6–97.0)	72.2 (51.5–92.9)	0.705
Specificity, % (95% CI)	92.8 (90.2–95.4)	75.5 (71.2–79.8)	<0.001	85.6 (78.3–92.8)	75.6 (66.7–84.4)	0.0495
PPV, % (95% CI)	36.4 (22.1–50.6)	15.2 (8.5–21.8)	<0.001	51.9 (33.0–70.7)	37.1 (21.1–53.2)	0.073
NPV, % (95% CI)	98.4 (97.1–99.7)	98.3 (96.9–99.8)	0.966	95.1 (90.3–99.8)	93.2 (87.4–98.9)	0.562
Detection of CIN2+						
Sensitivity, % (95% CI)	49.0 (35.3–62.7)	62.7 (49.5–76.0)	0.108	73.3 (57.5–89.2)	66.7 (49.8–83.5)	0.527
Specificity, % (95% CI)	94.7 (92.4–97.0)	77.7 (73.4–82.0)	<0.001	93.6 (88.2–99.0)	80.8 (72.0–89.5)	0.018
PPV, % (95% CI)	56.8 (42.2–71.5)	28.6 (20.2–36.9)	<0.001	81.5 (66.8–96.1)	57.1 (40.7–73.5)	0.011
NPV, % (95% CI)	92.9 (90.3–95.5)	93.6 (90.9–96.4)	0.553	90.1 (83.6–96.6)	86.3 (78.4–94.2)	0.307

a Calculated using McNemar's Chi-squared test. Abbreviations: CI, confidence interval; CIN, cervical intraepithelial neoplasia; HPV, human papillomavirus; No., number; NPV, negative predictive value; PPV, positive predictive value; TCT, Thinprep cytologic test.

Subsequently, 108 HPV16/18-positive women were selected for further analysis for the better triage capacity of HPV integration (Table 1). Of note, the superiority of HPV integration over TCT was prominent for detecting CIN3+, with marginally higher specificity (85.6% [78.3%–92.8%] vs. 75.6% [66.7%–84.4%], *P* = 0.0495), comparable sensitivity (77.8% [58.6%–97.0%] versus 72.2% [51.5%–92.9%], *P* = 0.705) (Table 1), indicating remarkable potential of HPV integration as a triage for CIN3+ in HPV16/18-positive patients. Comparable strengths including sensitivity, specificity, PPV, and NPV were observed in HPV integration for identification of CIN2+.

When stratified by age, similar results were observed in both women older and younger than 45 years (Supplementary Table 3). In each group, HPV integration demonstrated comparable sensitivity and significantly higher specificity for the diagnosis of CIN3+ compared with TCT, and the HPV integration testing strategy yielded a lower colposcopy referral rate than TCT testing strategy (both *P* < 0.05).

### 3.3 The immediate risk of CIN2+/CIN3+ by baseline HPV integration status and TCT results in HPV-positive women

**Fig. 2:**
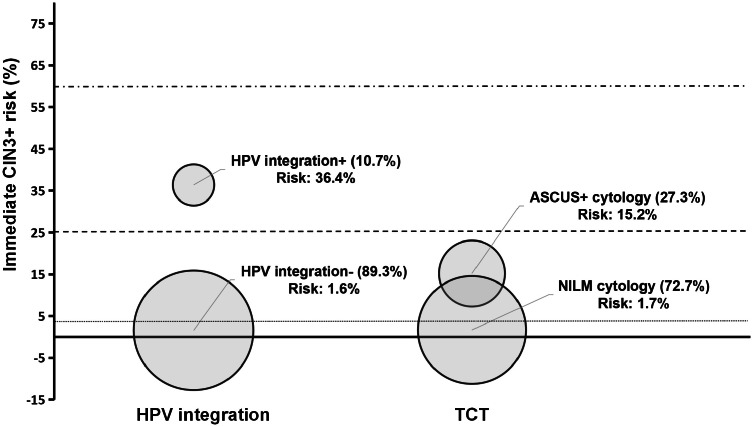
The immediate risk of CIN3+ for women stratified by HPV integration and cytology in HPV-positive women. The risks of CIN3+ are plotted on the y-axis, with the number and percentage of women indicated. The dotted line corresponds to the colposcopy referral threshold (4%). The dashed line corresponds to the expedited treatment or colposcopy acceptable threshold (25%). The dash-dotted line corresponds to the expedited treatment preferred threshold (60%). Abbreviations: ASCUS, atypical squamous cells of undetermined significance; CIN, cervical intraepithelial neoplasia; HPV, human papillomavirus; NILM, negative for intraepithelial lesion or malignant neoplasm.

In the baseline cross-sectional comparison analysis, the HPV integration-positive group exhibited the highest immediate risk for CIN2+ (56.8%) and CIN3+ (36.4%) when compared with the ASCUS+ group (28.6% for CIN2+, 15.2% for CIN3+) in HPV-positive participants (Supplementary Table 4, Fig. 2, and Supplementary Fig. 2). More importantly, the proportion of HPV integration-negative patients is significantly higher than that of the NILM group (89.3% [366/410] vs. 72.7% [298/410], *P* < 0.001), with comparable immediate risk of CIN3+ (1.6% vs. 1.7%). (Supplementary Table 4, Fig. 2 and Supplementary Fig. 2). When stratified by age, the advantage of HPV integration testing in triage is more pronounced in the population older than 45 years (Supplementary Table 4). For women aged ≥45 years, HPV integration exhibited higher specificity and PPV than TCT in diagnosing CIN3+, reducing false positives and thus avoiding unnecessary colposcopies while alleviating both psychological and economic burdens for patients.

Next, women with HPV16/18 infection were selected as a high-risk group for independent analysis (Table 2). Stratified by HPV integration and cytology, the lowest immediate risk of CIN2+ or CIN3+ was observed in the HPV integration-negative group (9.9% for CIN2+, 4.9% for CIN3+), which was lower than those in the normal cytology group (13.7% for CIN2+, 6.8% for CIN3+). The highest immediate risks of CIN2+ or CIN3+ were observed in the HPV integration-positive group (Table 2). These results indicated that HPV integration may be of remarkable value as a triage biomarker for the HPV16/18-positive population.

**Table 2:** The immediate risk stratification by HPV integration and cytology in HPV16/18-positive women.

HPV infection type	Cases	Immediate CIN2+ risk, % (No./total patients)	Immediate CIN3+ risk, % (No./total patients)
HPV integration	108	27.8 (30/108)	16.7 (18/108)
Integration (+)	27	81.5 (22/27)	51.9 (14/27)
Integration (-)	81	9.9 (8/81)	4.9 (4/81)
TCT			
≥ASCUS	35	57.1 (20/35)	37.1 (13/35)
NILM	73	13.7 (10/73)	6.8 (5/73)

Abbreviations: ASCUS, atypical squamous cells of undetermined significance; CIN, cervical intraepithelial neoplasia; HPV, human papillomavirus; NILM, negative for intraepithelial lesion or malignant neoplasm; No., number; TCT, Thinprep cytologic test.

### 3.4 The prognosis of patients with varying durations of HPV integration

Among the HPV-positive patients without abnormal pathological diagnoses at baseline, there were 47 women with transient HPV integration and 5 women with persistent HPV integration. The 5-year cumulative detection of CIN2+ in the persistent integration group was 20.0%, slightly higher than that in the transient integration positive group but with no significant difference (4.3%) (*P* = 0.27, Supplementary Table 5).

## 4 Discussion

Our results showed that HPV integration might be a promising triage strategy for HPV-positive patients. By focusing on 410 hrHPV-positive women, we found that the HPV integration sensitivity among CIN3+ patients reached 72.7%, which was comparable to TCT. Moreover, the specificity of HPV integration for the detection of CIN3+ (92.8%) was significantly higher than TCT (75.5%) (*P* < 0.001). HPV integration testing strategy also yielded a significantly lower colposcopy referral rate than TCT testing strategy (10.7% vs. 27.3%, *P* < 0.001). In our study, HPV integration testing exhibited better risk stratification for HPV16/18-positive women compared with cytology testing. Therefore, our results suggest HPV integration status could serve as a surrogate triage biomarker for the detection of cervical precancers, as well as the HPV16/18-positive population.

International guidelines currently recommend HPV testing as a primary screening method for cervical cancer.^30^ Cytology, a traditional screening method used to detect cervical cell morphologies, is often recommended as a triaging method when HPV is detected as positive.^24^ However, it is worth noting that the natural regression rate is 50% in the population with CIN2+ at follow-up. Among women under 30 years of age, the regression rate is as high as 66%.^31^^,^^32^ Hence, we chose CIN3+ as the main endpoint instead of CIN2+, as it was widely considered closer to cervical cancer and was a more valuable endpoint for assessing the risk of cervical precancerous lesions.^33^ In this study, women older than 45 years old with positive HPV integration exhibited a higher immediate risk of CIN3+ (40.7%) than women under 45 years (29.4%). This increased risk may be partially attributed to a relative deficiency in viral clearance and insufficient adaptive immune responses in women over 45 years of age.^34^

However, there were no differences between women in different age groups with abnormal TCT tests (Supplementary Table 4). Therefore, using TCT as a triaging method for HPV-positive individuals is bound to cause excessive colposcopy examination and invasive biopsy.

The concept of health management has gained widespread acceptance in recent years, with a focus on disease prevention and control as a cornerstone of modern health theory. The continuous accumulation of risk over time is driven by positive feedback effects associated with pathogenic factors. Once the risk accumulates to a certain threshold, qualitative changes occur within the lesion. The classic example is the process of HPV infection developing into cervical precancerous lesions and then to cervical cancer. HPV infection is widely recognized as the cause of cervical cancer, typically taking 5 to 15 years for HPV infection to progress to cervical cancer.^35^ The occurrence of cervical cancer is an occasional event following HPV infection. Most HPV infections are transient, and it is only when the HPV oncogenes integrate into the host genome and form continuous infection in cervical epithelial cells that this is believed to be the key molecular basis for the development of cervical cancer.^36^^,^^37^

HPV integration plays a crucial role in cervical carcinogenesis and is regarded as a hallmark of HPV-induced cancer development.^16^^,^^38^ In our previous study, eleven high-frequency HPV integration sites were detected in cervical cancer and precancerous tissues.^26^ The integration process induces various oncogenic effects on host cells. Insertional events may cause genomic instability,^39^ triggering mutations in critical cancer-associated genes and potentially leading to the malignant transformation of infected cervical epithelial cells.^40^^,^^41^ Additionally, integrated viral elements could function as *cis*-activators of adjacent oncogenes, thereby inducing carcinogenesis.^42^ Furthermore, viral integration may produce virus-human fusion transcripts/proteins that might act as carcinogenic drivers, providing host cells with additional selective advantages in transformation for host cells.^41^^,^^43^ Recently, through large-scale, multi-omics (clinical, genomic, transcriptional, proteomic, phosphoproteomic, and single-cell) data,^28^ we have demonstrated that silent and productive HPV integration may play different roles in the development of cervical cancer. Tumors with productive HPV integration exhibit elevated levels of E6/E7 proteins and increased tumor aggressiveness and immunoevasion. Productive HPV integration is under selection and likely contributes to cervical cancer pathophysiology. Besides, antivirus immune responses have been proposed to enhance HPV clearance, with potential selection for cells with HPV integration. In the present study, we demonstrated that some HPV integrations can be cleared and persistent HPV integration carries a higher risk of disease than transient HPV integration, which supports this hypothesis.

To our knowledge, this is the first population-based, prospective, clinical observation study focusing on the performance of HPV integration as a triage tool in cervical lesion screening. Limited studies were conducted in analyzing the relationship between HPV integration and cervical lesions in populations. This study includes cross-sectional and longitudinal analyses based on a 5-year follow-up to assess the impact of HPV integration on cervix lesion progress. With the support of more clinical data, HPV integration holds promise as an objective, sensitive, and practical triage strategy for cervical cancer screening.

Our study demonstrated that HPV integration testing exhibited better risk stratification for HPV-positive women compared with cytology testing. Besides, our results suggest HPV integration status could serve as a surrogate triage biomarker for the detection of cervical precancers in HPV-positive women, as well as the HPV16/18-positive population. To facilitate the translation into clinical application, multicenter prospective studies with larger sample sizes are currently still required.

The main limitation of this study is the relatively small sample size due to the lower HPV infection rate and HPV integration rate in the population-based setting compared with the hospital-based setting. Although our data demonstrated the advantages of HPV integration as a triage method, limited observable cases prevented some comparative data from achieving statistical significance. Therefore, future studies with increased sample sizes are necessary.

In conclusion, HPV integration is the key molecular basis of persistent HPV infection, promoting cervical lesions that eventually progress to cervical cancer. HPV integration may better stratify the immediate risk of cervical lesions. Therefore, the HPV integration test holds promise as a means of triage for HPV-positive women in preliminary screening.

## Declaration of competing interest

Ding Ma and Zheng Hu are inventors of a patent (ZL201510217845.0) for the HPV integration test. All other authors declare that they have no known competing financial interests or personal relationships that could have appeared to influence the work reported in this paper.

## Appendix Supplementary materials

Image, application 1
