# Supplementary material for: Risk assessment and triage strategy of cervical cancer primary screening on HPV integration status: 5-year follow-up of a prospective cohort study
Source: J Natl Cancer Cent. 2024 Oct 16;4(4):311–7. doi: 10.1016/j.jncc.2024.08.001 (PMC11674434; doi:10.1016/j.jncc.2024.08.001)
Supplement: Supplementary file 1 [file mmc1.pdf]

Risk assessment and triage strategy of cervical cancer primary screening on HPV integration status: 5-year follow-up of a prospective cohort study

Xun Tian, Danhui Weng, Ye Chen, Yi Wang, Xiao Li, Xin Wang, Chen Cao, Danni Gong, Zhen Zeng, Qiongyan Wu, Xueqian Wang, Peng Wu, Lu Fan, Qinghua Zhang, Hui Wang, Zheng Hu, Xiaodong Cheng, Ding Ma

Supplementary Materials

Supplementary Table 1. Characteristics of the participants at baseline.

Characteristic	2015 (N=4086) No./Total patients (%)
**Age, years**	
≤45	1821/4086 (10.0)
>45	2265/4086 (90.0)
**TCT**	
NILM	3866/4086 (94.6)
≥ASCUS	220/4086 (5.4)
ASCUS	76/4086 (1.9)
ASC-H	17/4086 (0.4)
LSIL	100/4086 (2.4)
HSIL	18/4086 (0.4)
AGC	5/4086 (0.1)
AIS	0
Cancer	4/4086 (0.1)
**HPV**	
Negative	3676/4086 (90.0)
Positive	410/4086 (10.0)
HPV16(+) or HPV18(+)	108/4086 (1.3)
HPV integration	27/108 (25)
HPV non-integration	81/108 (75)
Other types(+)	302/4086 (7.4)
HPV integration	17/302 (5.6)
HPV non-integration	285/302 (94.4)
**Pathology**	
Normal	3966/4086 (97.1)
Abnormal	120/4086 (2.9)
CIN1	67/4086 (1.6)
CIN2	31/4086 (0.8)
CIN3	20/4086 (0.5)
Cancer	2/4086 (0.05)

Abbreviations: AIS, adenocarcinoma in situ; AGC, atypical glandular cells; ASC-H, atypical squamous cells – cannot exclude high-grade squamous intraepithelial lesion; ASCUS, atypical squamous cells of undetermined significance; HPV, human papillomavirus; HSIL, high-grade squamous intraepithelial lesion; LSIL, low-grade squamous intraepithelial lesion; NILM, negative for intraepithelial lesion or malignant neoplasm; TCT, Thinprep cytologic test.

Supplementary Table 2. Relationship between HPV integration, TCT, and pathology.

	HPV Integration				TCT			
	Total	Positive	Positive rate	*P* value	Total	≥ ASCUS	%	*P* value
Cancer	2	2	100%	<0.001	2	1	50%	<0.001
CIN3	20	14	70%		20	16	80%	
CIN2	29	9	31%		29	15	51.7%	
CIN1	58	4	6.9%		58	23	39.7%	
Normal	301	15	5.0%		301	57	18.9%	

Supplementary Table 3. Triage performance for detection of CIN2+/CIN3+ in HPV-positive women stratified by age.

	HPV-positive women (age >45 years old)			HPV-positive women (age ≤45 years old)		
	**HPV Integration**	**TCT**	***P* value**	**HPV Integration**	**TCT**	***P* value**
**Positivity, No./Total patients**	10.5%, 27/257	24.1%, 62/257	<0.001	11.1%, 17/153	32.7%, 50/153	<0.001
**Colposcopy referral rate, No./Total patients**	10.5%, 27/257	24.1%, 62/257		11.1%, 17/153	32.7%, 50/153	
**Detection of CIN3+**						
Sensitivity, % (95% CI)	84.6 (65.0–100.0)	76.9 (54.0–99.8)	0.655	55.6 (23.1–88.0)	77.8 (50.6–100.0)	0.317
Specificity, % (95% CI)	93.4 (90.3–96.5)	78.7 (73.6–83.8)	<0.001	91.7 (87.2–96.2)	70.1 (62.7–77.6)	<0.001
PPV, % (95% CI)	40.7 (22.2–59.3)	16.1 (7.0–25.3)	0.002	29.4 (7.8–51.1)	14.0 (4.4–23.6)	0.096
NPV, % (95% CI)	99.1 (97.9–100.0)	98.5 (96.7–100.0)	0.531	97.1 (94.2–99.9)	98.1 (95.4–100.0)	0.528
**Detection of CIN2+**						
Sensitivity, % (95% CI)	59.3 (40.7–77.8)	55.6 (36.8–74.3)	0.705	37.5 (18.1–56.9)	70.8 (52.6–89.0)	0.021
Specificity, % (95% CI)	95.2 (92.5–98.0)	79.6 (74.4–84.8)	<0.001	93.8 (89.6–98.0)	74.4 (66.9–81.9)	<0.001
PPV, % (95% CI)	59.3 (40.7–77.8)	24.2 (13.5–34.9)	<0.001	52.9 (29.2–76.7)	34.0 (20.9–47.1)	0.094
NPV, % (95% CI)	95.2 (92.5–98.0)	93.8 (90.5–97.2)	0.267	89.0 (83.7–94.2)	93.2 (88.3–98.1)	0.095

Abbreviations: CIN, cervical intraepithelial neoplasia; HPV, human papillomavirus; NILM, negative for intraepithelial lesion or malignant neoplasm; NPV, negative predictive value; PPV, positive predictive value; TCT, Thinprep cytologic test.

Supplementary Table 4. The immediate risk stratification using HPV integration and cytology in 410 HPV-positive women aged ≤45 and > 45 years.

	**All (N=410)**		**≤45 years (N=153)**		**>45 years (N=257)**	
	**Immediate CIN2+ risk**	**Immediate CIN3+ risk**	**Immediate CIN2+ risk**	**Immediate CIN3+ risk**	**Immediate CIN2+ risk**	**Immediate CIN3+ risk**
**Risk stratification by age**	12.4% (51/410)	5.4% (22/410)	15.7% (24/153)	5.9% (9/153)	10.5% (27/257)	5.1% (13/257)
**HPV integration**						
**Positive**	56.8% (25/44)	36.4% (16/44)	52.9% (9/17)	29.4% (5/17)	59.3% (16/27)	40.7% (11/27)
**Negative**	7.1% (26/366)	1.6% (6/366)	11.0% (15/136)	2.9% (4/136)	4.8% (11/230)	0.9% (2/230)
**TCT**						
**≥ASCUS**	28.6% (32/112)	15.2% (17/112)	34% (17/50)	14% (7/50)	24.2% (15/62)	16.1% (10/62)
**NILM**	6.4% (19/298)	1.7% (5/298)	6.8% (7/103)	1.9% (2/103)	6.2% (12/195)	1.5% (3/195)

Abbreviations: ASCUS, atypical squamous cells of undetermined significance or worse; CIN, cervical intraepithelial neoplasia; HPV, human papillomavirus; NILM, negative for intraepithelial lesion or malignant neoplasm; TCT, Thinprep cytologic test.

Supplementary Table 5. Accumulative detection of CINs according to HPV integration status.

Pathology	Transient HPV integration (N=47)	Persistent HPV integration (N=5)	*P* value
CIN3+	0% (0/47)	0% (0/5)	>0.99
CIN2+	4.3% (2/47)	20% (1/5)	0.27

Note: Women with only once detected for HPV integration were defined as transient HPV integration. Women with ≥ twice detected for HPV integration were defined as persistent HPV integration. In the 2020 follow-up, the accumulative detection of CIN is presented for patients with HPV integration at baseline. Abbreviations: CIN, cervical intraepithelial neoplasia; HPV, human papillomavirus.

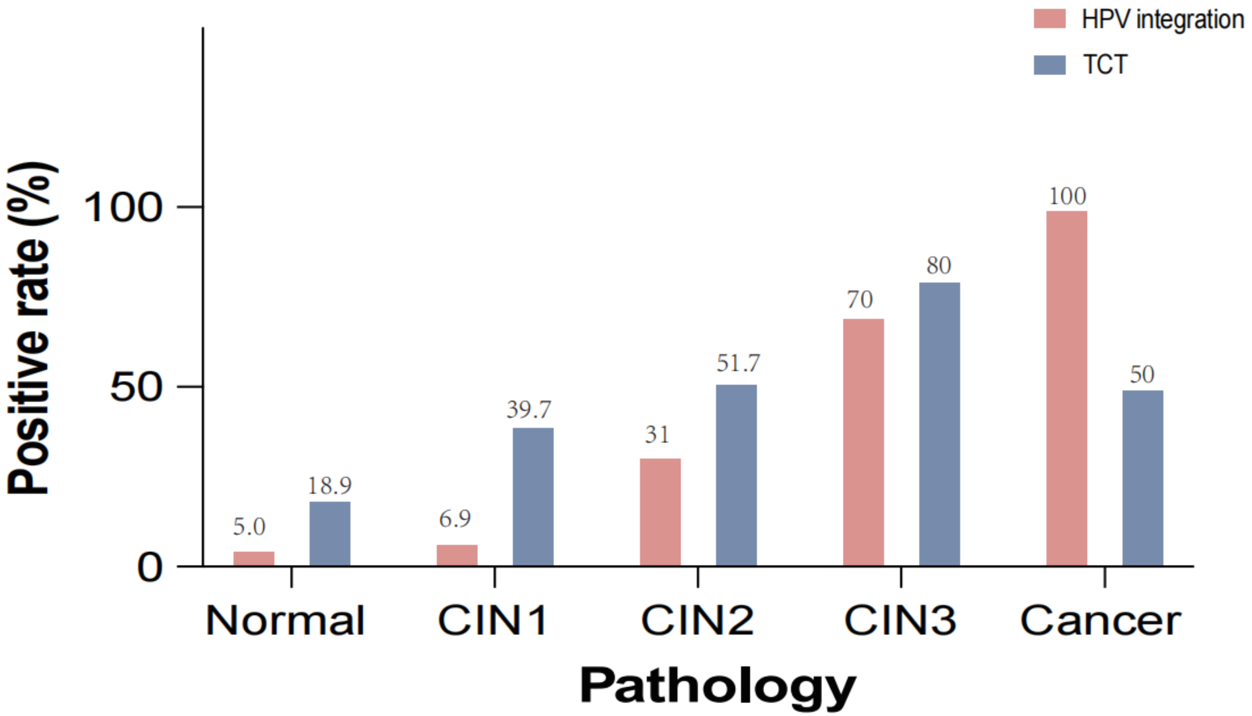

Supplementary Fig. 1. Trend in the positive rates of HPV Integration and TCT by CIN. CIN, cervical intraepithelial neoplasia; HPV, human papillomavirus; TCT, Thinprep cytologic test.

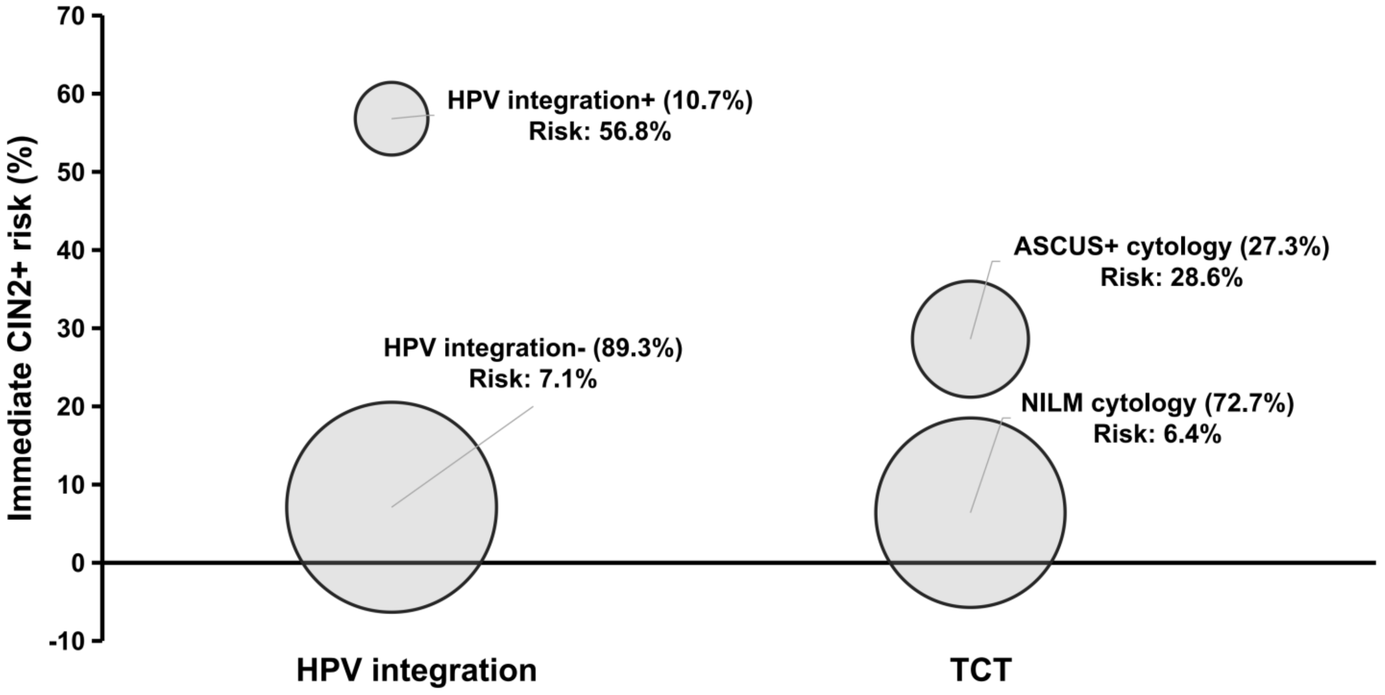

Supplementary Fig. 2. The immediate risk of CIN2+ for women stratified by HPV integration and cytology in HPV-positive women. The risks of CIN2+ are plotted on the y-axis, with the number and percentage of women indicated. ASCUS, atypical squamous cells of undetermined significance or worse; CIN, cervical intraepithelial neoplasia; HPV, human papillomavirus; NILM, negative for intraepithelial lesion or malignant neoplasm.
